# Cat Scratch Disease: An Unusual Case of Right Inguinal Lymphadenitis Due to Bartonella henselae

**DOI:** 10.7759/cureus.44280

**Published:** 2023-08-28

**Authors:** Zoheb I Sulaiman, Hasan Samra, Gina Askar

**Affiliations:** 1 Infectious Diseases, Augusta University Medical College of Georgia, Augusta, USA; 2 Pathology, Augusta University Medical College of Georgia, Augusta, USA

**Keywords:** inguinal lymphadenopathy, inguinal lymphadenitis, bartonella, bartonella henselae, cat-scratch disease

## Abstract

Cat scratch disease (CSD) is caused by a bacterial infection due to *Bartonella henselae* and is associated with young cats and kittens. CSD commonly occurs as regional lymphadenitis in the setting of subacute regional lymphadenopathy predominantly in children and young adults. The prognosis for immunocompetent patients is favorable with complete recovery, however, immunocompromised adults can progress to life-threatening complications such as neuroretinitis, osteomyelitis, and bacillary angiomatosis. *B. henselae* is transmitted from cats to humans through scratching or biting when located on the cat’s claws or oral cavity. In 1% of diagnosed cases, patients developed this disease without ever receiving an animal scratch.

We present a case of a 29-year-old immunocompetent male developing severe right inguinal pain with concern for an incarcerated inguinal hernia. He reported exposure to a vaccinated six-month-old kitten but denied any recent scratches or bites. His infectious workup revealed right inguinal lymphadenitis on CT imaging and subsequent lymph node biopsy confirmed a diagnosis of CSD. He was treated with a short course of oral doxycycline for CSD and opioids for pain management. This case illustrates the importance of thorough complete history and physical taking even in immunocompetent patients and early recognition with prompt targeted treatment of *Bartonella* lymphadenitis to prevent unfavorable outcomes.

## Introduction

*Bartonella henselae* is a Gram-negative, facultative intracellular bacillus that can be transmitted to humans via scratches or bites from exposure to cats or cat fleas [1-5]. It can also be spread via contact with cat saliva through a person's broken skin or mucosal surfaces. *Bartonella* infections range from self-limited to severe cases based on a patient’s immune status. Clinical manifestations include cat scratch disease (CSD), bacteremia, periadenitis, bacillary angiomatosis, endocarditis, osteomyelitis, and neuroretinitis [6-7]. 

CSD occurs with symptoms of self-limited fever and subacute, tender, regional lymphadenopathy near the site of bacterial inoculation after being scratched or bitten by a carrier cat [5]. The inoculation sites (head/neck, chest, hands, and arms) usually progress to a primary granulomatous lesion 3 to 10 days after inoculation; this condition most commonly involves the cervical (26%) and axillary (46%) lymph nodes and inguinal (17%) localization has been rarely described [3,5].

In 2016, Nelson et al. conducted a retrospective study of patients younger than 65 years of age and identified 13,273 patients with a diagnosis of CSD from 2005 to 2013 in the United States [6]. In this analysis, the incidence of atypical CSD (involving the eye, heart, liver, spleen, etc.) accounts for 1.5% of all cases, resulting in an average annual incidence of 0.7 cases/100,000 population and occurred most commonly in individuals 15 to 49 years of age.

Laboratory diagnosis of CSD is based on histologic findings of tissue or lymph nodes, detection of antibodies to *B. henselae* in the serum, and PCR assays of tissue [6,8]. CSD should be considered in immunocompetent adults with unilateral lymphadenitis, particularly with exposure to cats even without scratches or bites.

## Case presentation

**Figure 1:**
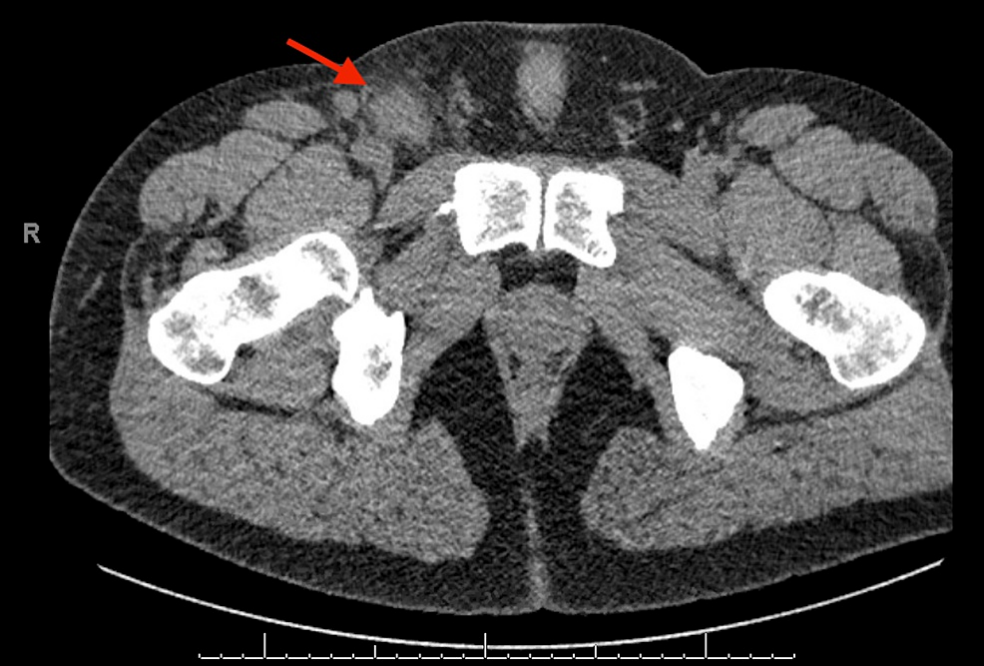
CT abdomen and pelvis (axial view) The red arrow shows enlarged right inguinal lymph nodes with surrounding inflammation concerning lymphadenitis.

**Figure 2:**
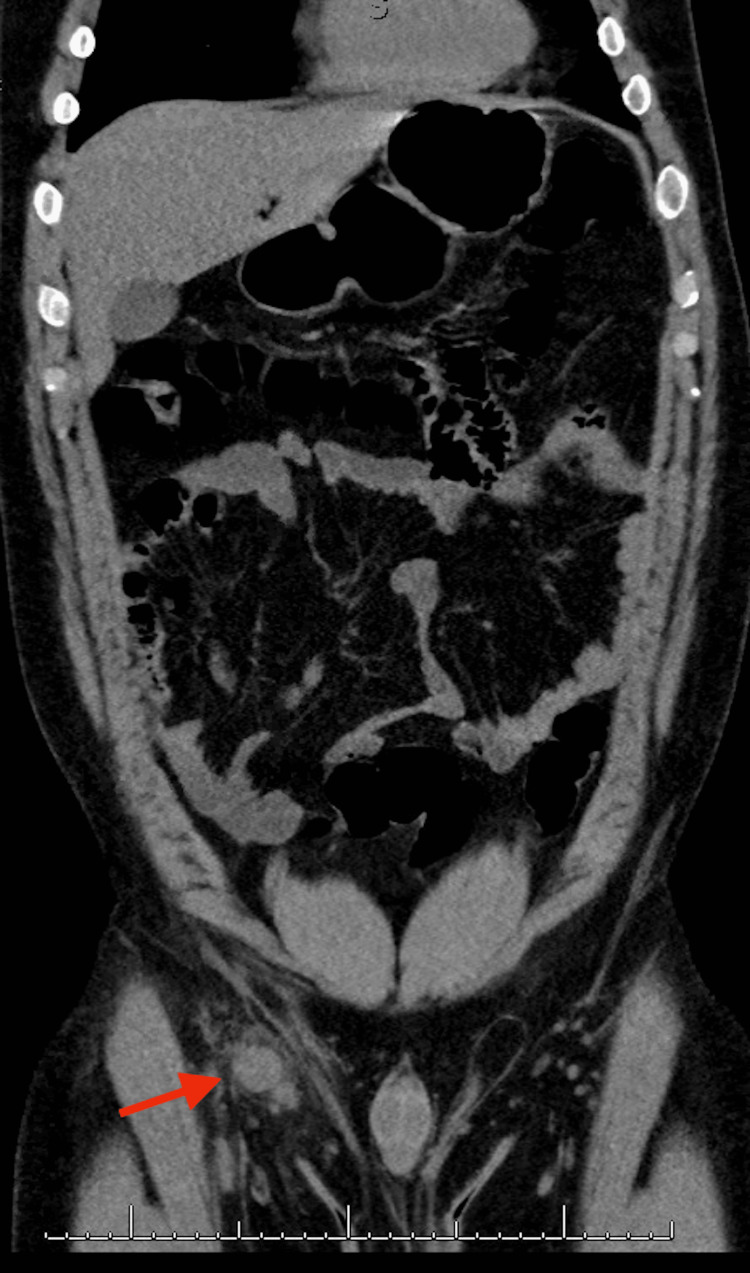
CT abdomen and pelvis (sagittal view) The red arrow shows right inguinal lymphadenitis suspicious of cat-scratch disease.

A 28-year-old male was referred to a surgery clinic for evaluation of a right inguinal hernia. He complained of a painful right groin bulge and generalized fatigue for five days. On triage, he was febrile to 38.4°C and tachycardic and the physical exam demonstrated painful right inguinal lymphadenopathy and red dome-shaped papules (one on the inguinal canal with minimal drainage and one along his lateral pelvis). He was hospitalized for further evaluation. CT imaging revealed multiple right inguinal lymph nodes with surrounding inflammation (max size 2.8 x 2.2 cm, seen in Figures 1, 2). Lab tests showed a white blood cell (WBC) count of 10.3, hemoglobin of 16.3, and platelet count of 181 with a WBC differential including 74% neutrophils, 17% lymphocytes, 8% monocytes, 1% eosinophils (Table 1), and unremarkable urinalysis. He was started on empiric ceftriaxone and azithromycin for right lymphadenitis. 

**Table 1:** Laboratory Parameters thous: Thousand

Laboratory Parameter	Levels at Presentation	Normal Range
White blood cell	10.3 thous/mm^3^	4.5-11.0 thous/mm^3^
Hemoglobin	16.3 g/dL	14.0-18.0 g/dL
Platelet Count	181 thous/mm^3^	150-400 thous/mm^3^
Neutrophils	74%	40-75%
Lymphocytes	17%	25-40%
Monocytes	8%	2-14%
Eosinophils	1%	0-5%

**Figure 3:**
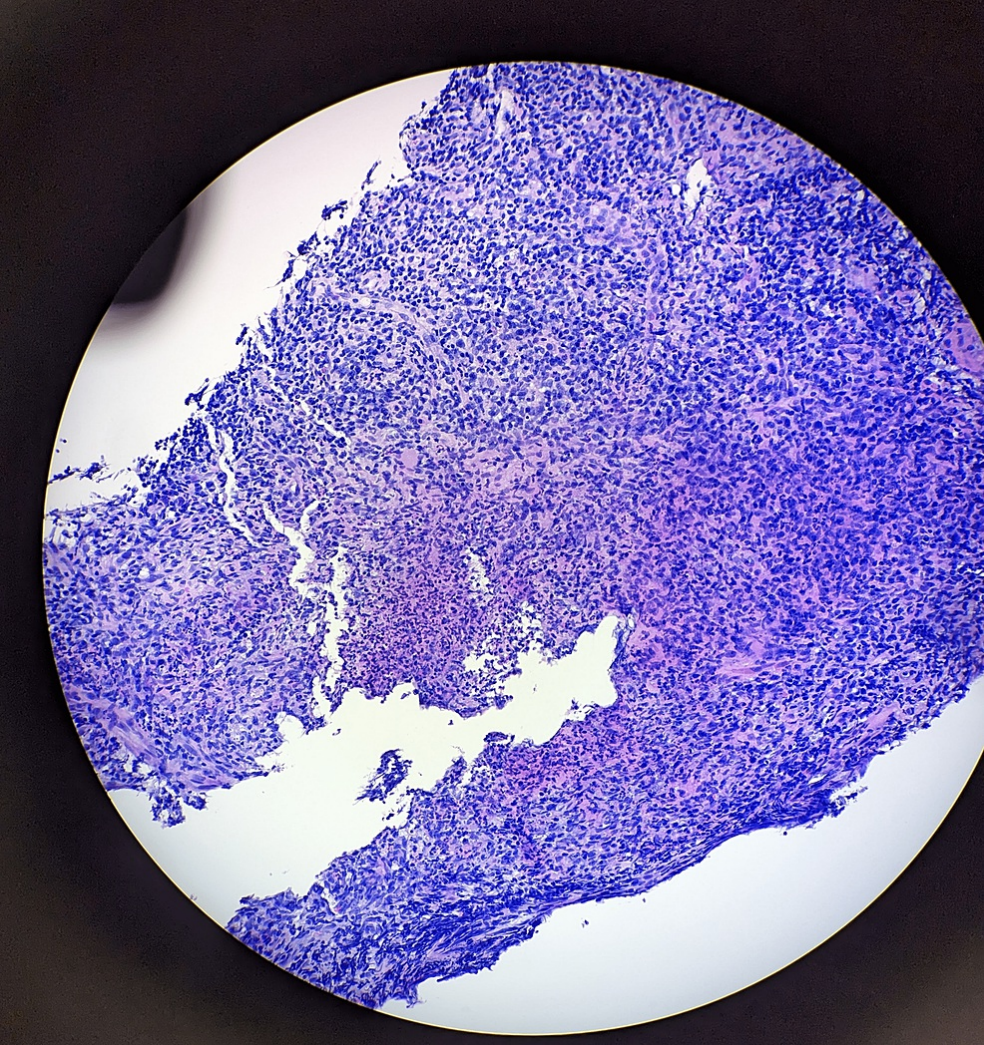
Lymph node biopsy result The biopsy image demonstrates stellate granuloma consistent with cat scratch disease (20X).

**Figure 4:**
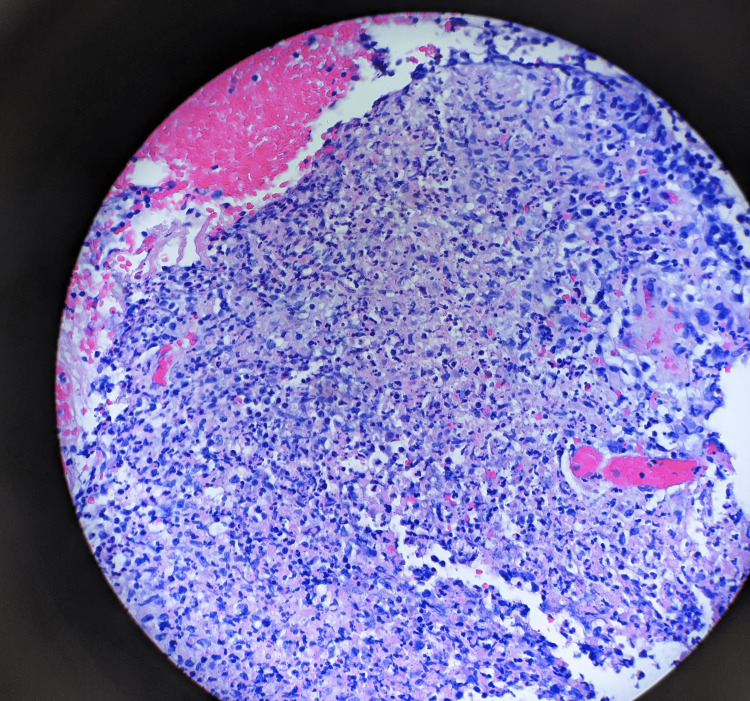
Lymph node biopsy result The image shows stellate granuloma with central necrosis, neutrophilic infiltration, and palisading histiocytes (100X).

On further questioning, he disclosed he was sexually active and monogamous with his female fiance. He had exposure to a six-month-old kitten but denied recent scratches or bites. Workup for sexually transmitted infections (STI) returned negative for syphilis, chlamydia, gonorrhea, trichomoniasis, and HIV. He underwent lymph node biopsy and both histopathology and broad-range 16S rRNA bacterial polymerase chain reaction (PCR) testing confirmed the diagnosis of CSD with *Bartonella* infection (seen in Figures 3, 4). Serologies for *B. henselae* immunoglobulin (Ig)M were positive at >1:20 and IgG positive at 1:4096 with negative blood cultures and flow cytometry excluding malignancy. He had an infusion reaction to azithromycin and was transitioned to doxycycline for a seven-day course and discharged home with oral opioids. Due to persistent inguinal pain, he went to an outside hospital a few months later and underwent a right inguinal lymph node dissection with complete resolution of symptoms.

## Discussion

The incidence of CSD in the US is 4.5-9.3 outpatient diagnoses per 100,000 people and 0.19-0.86 hospital admissions per 100,000 people [3]. A diagnosis of CSD requires three of the following four criteria: (1) contact with a cat or the presence of a scratch or primary lesion of skin or eye, (2) positive CSD skin test, (3) negative lab tests for other causes of lymphadenopathy, and (4) characteristic histopathological findings in a lymph node biopsy specimen [8]. The isolation of *Bartonella* species in a culture is difficult because of its fastidious nature; PCR and serology are preferred. The sensitivity of PCR with lymph node tissue is 30-60% for CSD; however, if histology and serology are coupled with PCR, the sensitivity increases to 87% [4].

Our patient met the diagnosis of CSD through his cat exposure, negative flow cytometry and STI workup, positive *Bartonella* serology, lymph node biopsy, and PCR. In mild to moderate CSD cases, it resolves within a few months without antibiotic therapy. Treatment recommendations include azithromycin for five days, or doxycycline/erythromycin for 10 days to two months in bacillary angiomatosis and immunocompromised patients. 

In this case, additional history was needed which helped direct organism-specific testing which ultimately made the diagnosis. Clinicians should maintain a high index of suspicion for CSD when evaluating patients with unilateral lymphadenitis, even without a clear antecedent cat-related injury or trauma.

## Conclusions

CSD can occur in individuals of any age and is no longer considered a disease in children. Depending on the severity, immunocompromised individuals or patients with atypical manifestations of CSD may require inpatient admission for further workup. If lymphadenitis does not resolve or progressively worsens, a tissue biopsy is indicated to rule out malignancy and to definitely diagnose CSD.
